# Supplementary figures and images for: How to design an art-science program? Self-reported benefits for artists and scientists in the VI4 artist-in-residence program
Source: PLoS One. 2022 Dec 30;17(12):e0279183. doi: 10.1371/journal.pone.0279183 (PMC9803234; doi:10.1371/journal.pone.0279183)

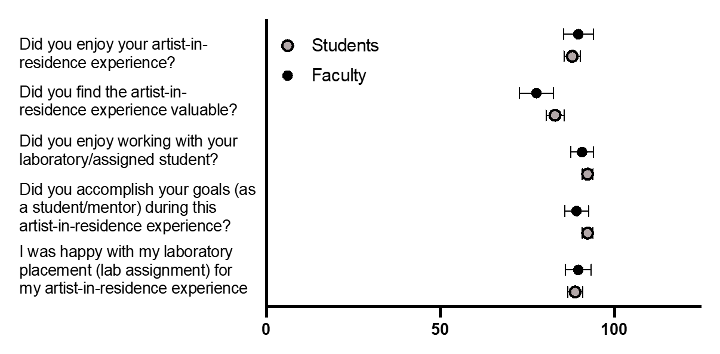

Supplement: S1 Fig — To determine if students and faculty responded differently to the prompts about their experience in the program, we graphed student and faculty responses identical to Fig 3 on the same graph. There was no significant difference between faculty and staff responses to survey questions. (PNG) [file pone.0279183.s002.png]

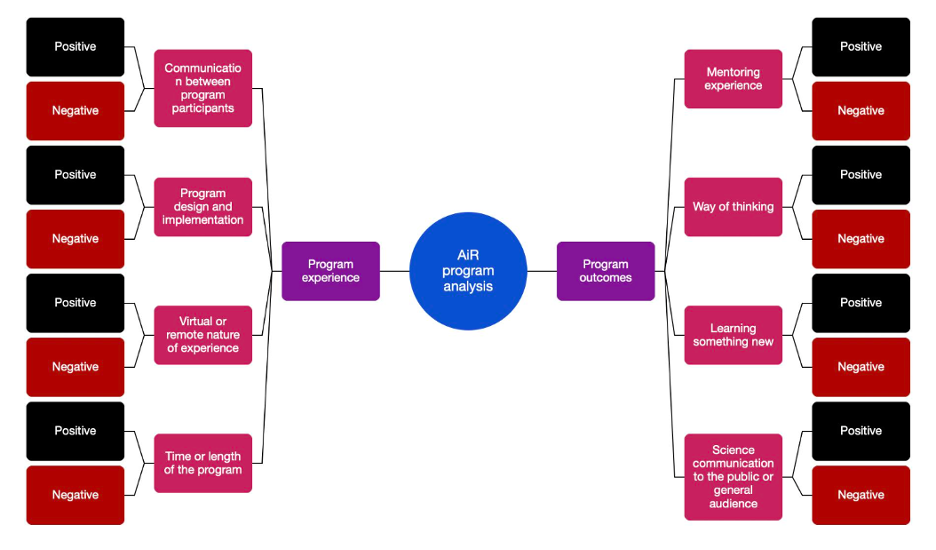

Supplement: S2 Fig — (PNG) [file pone.0279183.s003.png]

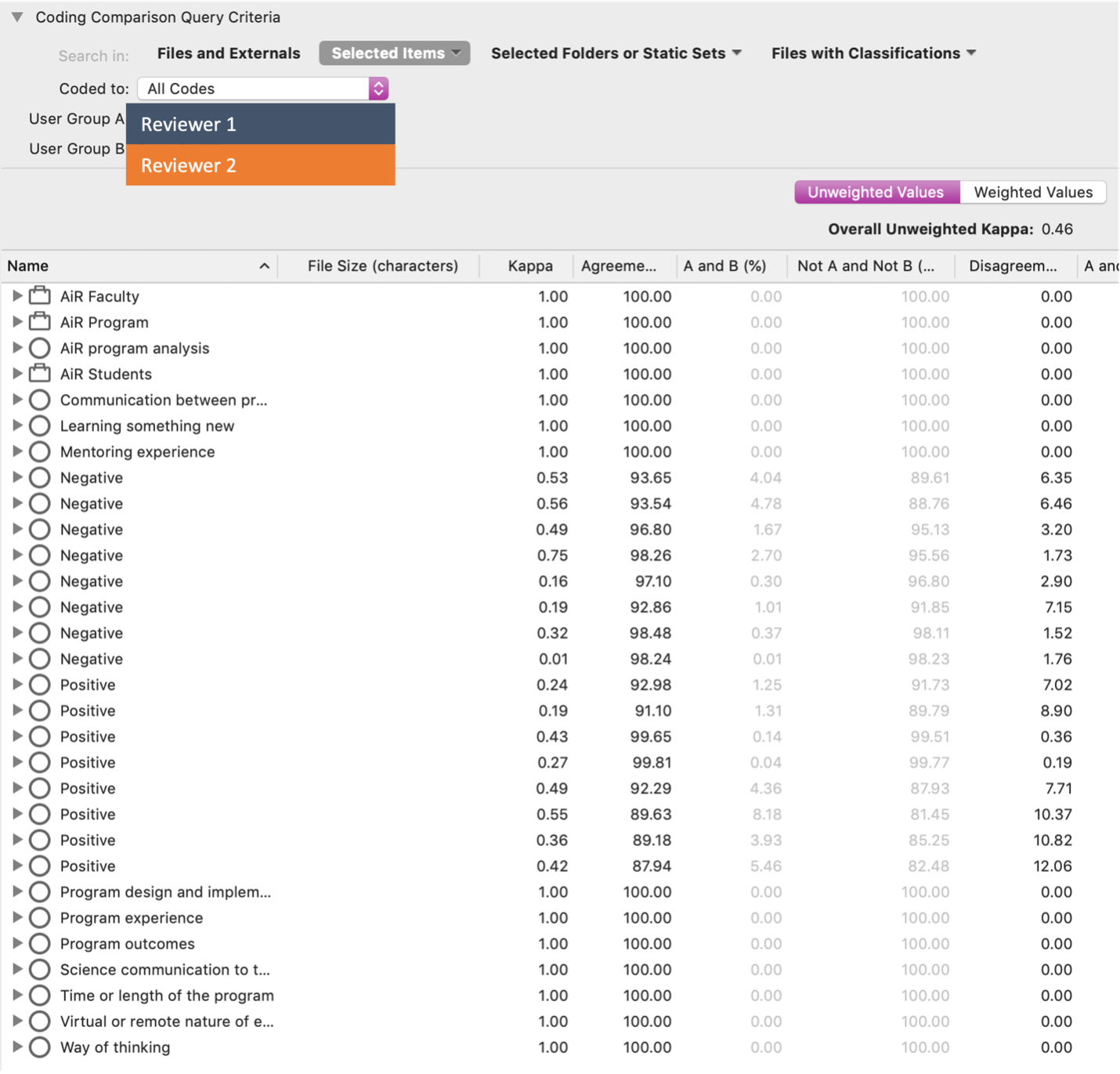

Supplement: S3 Fig — Available on Qualitative Data Repository (https://data.qdr.syr.edu/dataset.xhtml?persistentId=doi:10.5064/F6SUSRIC). (PNG) [file pone.0279183.s004.png]

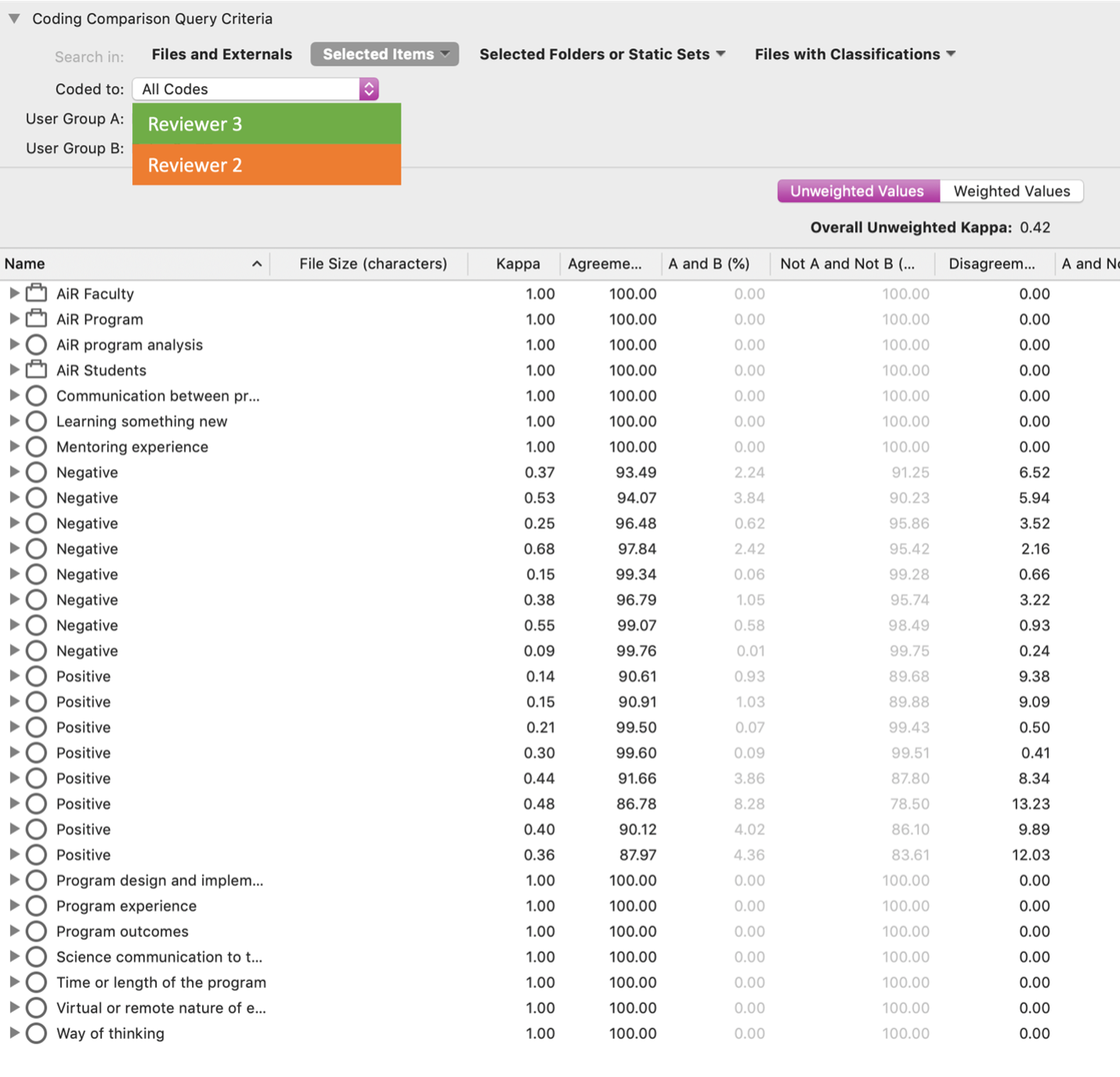

Supplement: S4 Fig — The NVivo files can be downloaded from the Qualitative Data Repository (https://data.qdr.syr.edu/dataset.xhtml?persistentId=doi:10.5064/F6SUSRIC). (PNG) [file pone.0279183.s005.png]

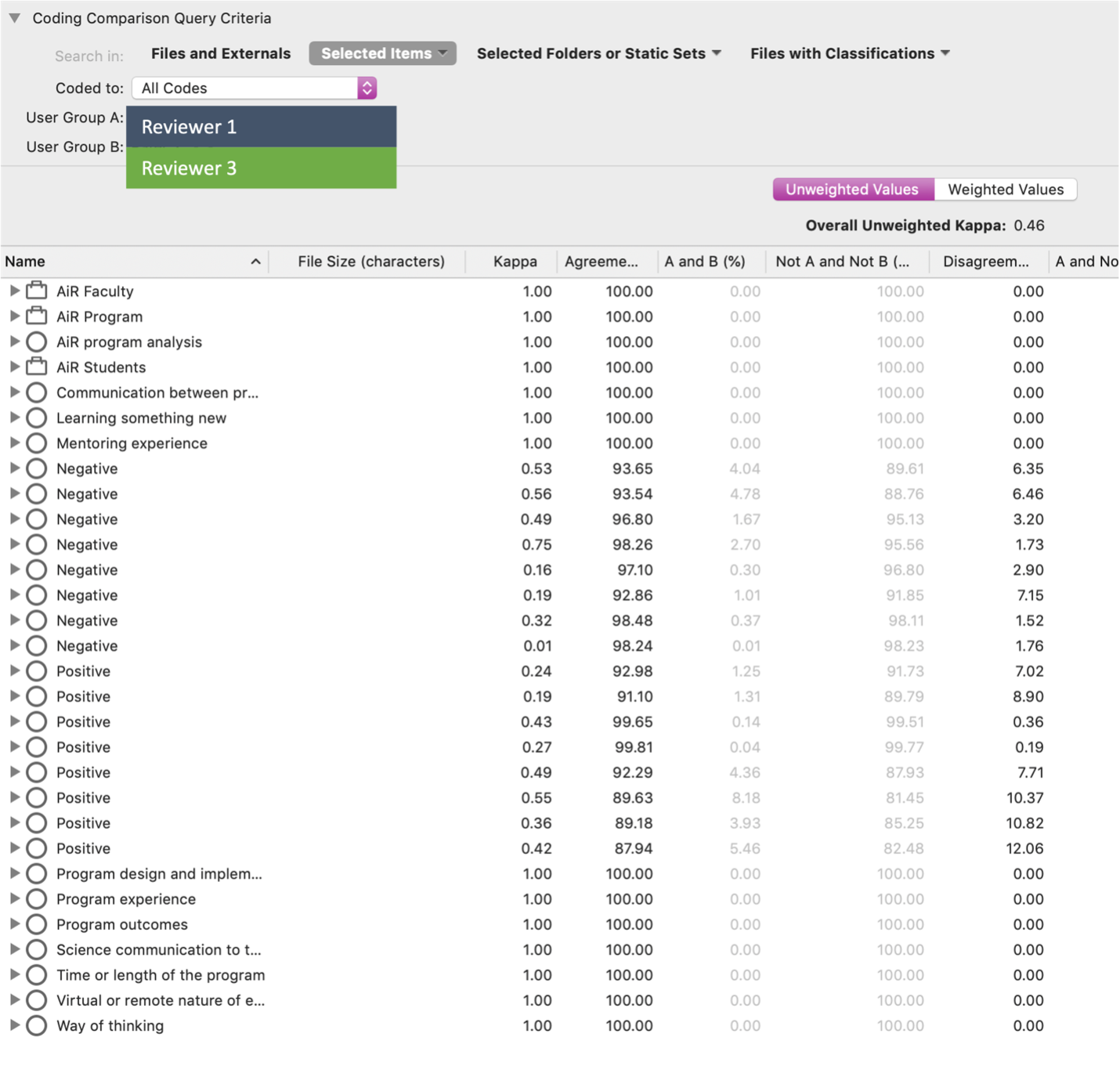

Supplement: S5 Fig — The NVivo files can be downloaded from the Qualitative Data Repository (https://data.qdr.syr.edu/dataset.xhtml?persistentId=doi:10.5064/F6SUSRIC). (PNG) [file pone.0279183.s006.png]

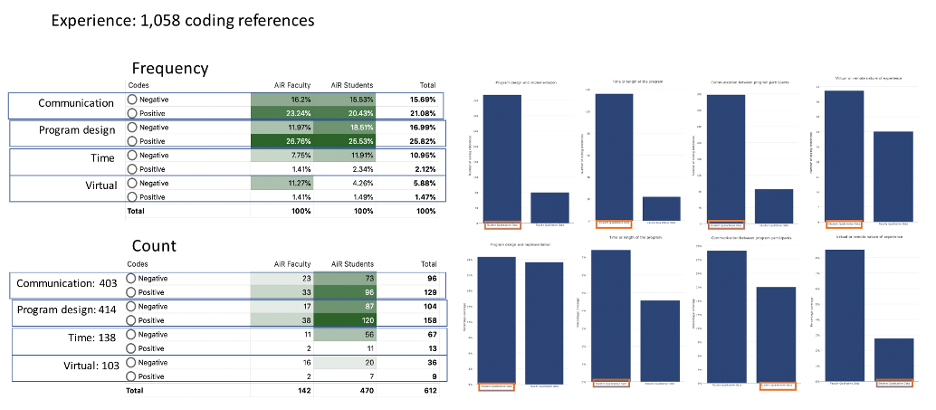

Supplement: S6 Fig — The NVivo files can be downloaded from the Qualitative Data Repository (https://data.qdr.syr.edu/dataset.xhtml?persistentId=doi:10.5064/F6SUSRIC). (PNG) [file pone.0279183.s007.png]

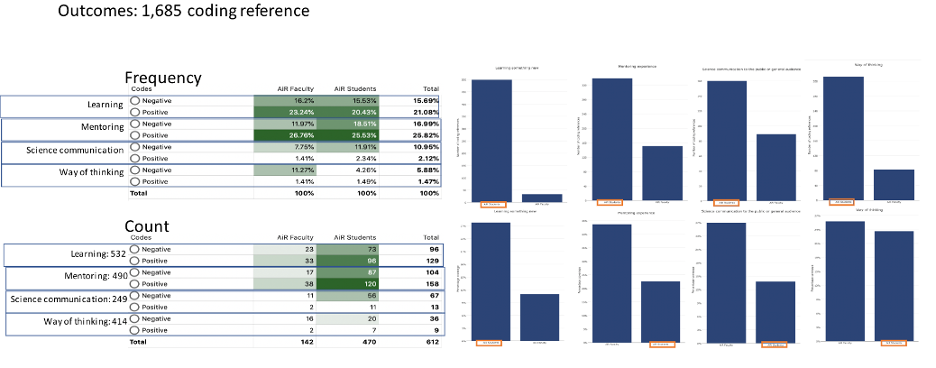

Supplement: S7 Fig — The NVivo files can be downloaded from the Qualitative Data Repository (https://data.qdr.syr.edu/dataset.xhtml?persistentId=doi:10.5064/F6SUSRIC). (PNG) [file pone.0279183.s008.png]
