# Supplementary material for: How to design an art-science program? Self-reported benefits for artists and scientists in the VI4 artist-in-residence program
Source: PLoS One. 2022 Dec 30;17(12):e0279183. doi: 10.1371/journal.pone.0279183 (PMC9803234; doi:10.1371/journal.pone.0279183)
Supplement: Attachment — Submitted filename: Response to Reviewers.docx [file pone.0279183.s009.docx]

Reviewer #2: The subject of the article is incomprehensible. Whatever the title, I don't know what the manuscript will be about. It needs to be improved. Abstract for complete reconstruction. Sentence: "The value of science communication in engaging the public has been well established." It is completely unfounded. The authors did not justify the importance of the work or its purpose. Unexplained abbreviations are used. It is unknown when the research was conducted on which sample and what results were obtained. Introduction. This point needs some fine-tuning. The authors justify the validity of the research in an interesting way, but do not state whether their proposal differs from other studies, what is novel in the work, and what gap is filled by the manuscript. I also recommend that you briefly describe what the AiR program is and why this program is important in terms of the topic under consideration. In the introduction, I also recommend that you briefly discuss how the manuscript is structured. The methodology, results, and conclusion are the strengths of the work. A weak point, however, is a very poor review of the literature, which requires a thorough elaboration. Figures 2b, c, and 3a, bc in the work are completely illegible.

Author responses:

• The title has been updated to “How to design an art-science program?; self-reported benefits for artists and scientists in the VI4 Artist-in-Residence program”

• The abstract was revised to o “While many new programs bridge the arts and sciences, data-based examination of art-science program design can lead to more efficient programming. The Vanderbilt Institute for Infection, Immunology, and Inflammation Artist-in-Residence program is a virtual program that brings together undergraduate student “artists” and faculty-level “scientists” to generate science-art content. We have recruited over 80 artists and 50 scientists to collaborate in creating visual science communication content. Using self-reported data from both groups, we performed qualitative and quantitative analyses to define sources for negative and positive experiences specifically for artists and scientists. We also identify areas for improvement and key features for in producing a positive experience. We found that artists participants had more positive responses about “learning something new” from the program than scientists. We also found that for both the artists and the scientists the length of the program and the virtual nature were identified as key features that could be modified. However, the most surprising aspect of our analysis suggests that for both “way of thinking” and “science communication to the public or general audience,” were seen as significant beneficial gains for scientists compared to artists. We conclude this analysis with suggestions to enhance the benefits and outcomes of an art-science program and ways to minimize the difficulties, such as communication and collaboration, faced by participants and program designers.

• We specifically address the reviewers’ comments about justify the importance of the work or its purpose through the following sentences o “While many new programs bridge the arts and sciences, a data-based examination of art-science program design can lead to more efficient programming.”

• We found no unexplained abbreviations but have significantly revised to text for added clarity including: o We have reduced the usage of the AiR acronym o We simplified the text by primarily using the terms “artist” and “scientist”

• To address the comments “It is unknown when the research was conducted on which sample and what results were obtained” we did the following: o We added this sentence to the end of the introduction “We collected 2019-2021 post-survey data from artist and scientists from the VI4 AiR program. We used both both qualitative and quantitative approaches to determine what the benefits are for both scientists and artists, how to better design the program, and discuss best practices in program design.”

• To address the comments: “The authors justify the validity of the research in an interesting way, but do not state whether their proposal differs from other studies, what is novel in the work, and what gap is filled by the manuscript” we have done the following: o We have added this statement to the introduction, “We believe that answering the lingering question of benefits for artists and scientists, respectively, in art-science collaborations can provide insightful design directions to accentuate professional growth for both artists and scientists. “

• Based on the reviewer’s comment “I also recommend that you briefly describe what the AiR program is and why this program is important in terms of the topic under consideration.” We added this to the first paragraph of the introduction

• Based on the reviewer’s comment “In the introduction, I also recommend that you briefly discuss how the manuscript is structured.” We added an overview in the last paragraph of the introduction.

• Based on the reviewer’s comment “A weak point, however, is a very poor review of the literature, which requires a thorough elaboration.” We extensively restructured the introduction.

• The reviewer mentioned that “Figures 2b, c, and 3a, bc in the work are completely illegible” and therefore we have redesigned all of the figures.
